# A Mini-Review for Cancer Immunotherapy: Molecular Understanding of PD-1/PD-L1 Pathway & Translational Blockade of Immune Checkpoints

**DOI:** 10.3390/ijms17071151

**Published:** 2016-07-18

**Authors:** Yongshu Li, Fangfei Li, Feng Jiang, Xiaoqing Lv, Rongjiang Zhang, Aiping Lu, Ge Zhang

**Affiliations:** 1 Institute of Precision Medicine and Innovative Drug Discovery, Institute of Science and Technology, Hong Kong Baptist University, Haimen 226133, China; yongshuli000@163.com (Y.L.); jiangfeng@nbu.edu.cn (F.J.); lxqd1@126.com (X.L.); zhangrj920@163.com (R.Z.); 2 Institute of Integrated Bioinformedicine and Translational Science, Hong Kong Baptist University, Hong Kong, China; fayebalaba@live.com; 3 Institute of Research and Continuing Education, Hong Kong Baptist University, Hong Kong, China; 4 Institute for Advancing Translational Medicine in Bone & Joint Diseases, School of Chinese Medicine, Hong Kong Baptist University, Hong Kong, China; 5 Faculty of Materials Science and Chemical Engineering, The State Key Laboratory Base of Novel Functional Materials and Preparation Science, Ningbo University, Ningbo 315211, China; 6 College of Medicine, Jiaxing University, Jiaxing 314001, China

**Keywords:** Immunotherapy, PD-1 (Programmed cell death protein 1), PD-L1 (Programmed death-ligand 1), monoclonal antibodies (mAbs)

## Abstract

Interference of the binding of programmed cell death protein 1 (PD-1) and programmed death-ligand 1 (PD-L1) has become a new inspiring immunotherapy for resisting cancers. To date, the FDA has approved two PD-1 monoclonal antibody drugs against cancer as well as a monoclonal antibody for PD-L1. More PD-1 and PD-L1 monoclonal antibody drugs are on their way in clinical trials. In this review, we focused on the mechanism of the PD-1/PD-L1 signaling pathway and the monoclonal antibodies (mAbs) against PD-1 and PD-L1, which were approved by the FDA or are still in clinical trials. And also presented is the prospect of the PD-1/PD-L1 immune checkpoint blockade in the next generation of immunotherapy.

## 1. Introduction

In 2012, 14.1 million new cancer cases were diagnosed worldwide and 8.2 million cancer patients passed away and more than 32.6 million people were living with cancer (within 5 years of diagnosis). About 57% (approximately 8 million) of new arising cancer cases, 65% (approximately 5.3 million) deaths of cancer and 48% (approximately 15.6 million) of the 5-year prevalent cancer cases have taken place in underdeveloped areas [1]. Surgery, radiotherapy and chemotherapy have been the main approaches for treating cancer in previous decades. Since Dana R. Leach first proposed an immune checkpoint blockade in 1996 [2], the first immune checkpoint inhibitor was approved by the FDA in 2011. By 2013, the cover report of science showed the new weapon of immunotherapy against cancer and the new therapeutic approach of using immune checkpoint inhibitors as anticancer agents was a landmark innovation. Undoubtedly, PD-1(Programmed cell death protein 1)/PD-L1(Programmed death-ligand 1) became the brightest star in 2015, after president Carter’s tumor was cured using anti-PD-1 antibody.

At least two signaling pathways need to be stimulated for the activation of cytotoxic T lymphocytes (CTLs) in secondary lymphoid tissues: Firstly, the binding occurs between peptide-MHC (major histocompatibility complex) complex with the availability of antigen presenting cells (APCs) and T cell receptors (TCRs). Then, the B7 molecules on APC interact with the CD28 receptors of T cells. It is noticeable that some experimental data suggests that a third necessary signal (such as IL-12, Type I IFN and adjuvant) plays a very important role in determining the fate of naïve CD8 T cells, tolerance or full activation [3]. Although less well studied, there is evidences of naïve CD4 T cells requiring a cytokine-dependent ′signal 3′ for a productive response to Ag and may be complementary to IL-1 [4]. Cellular immunity mediated by T cell is strictly supervised and controlled by a check/balance system performed though many stimulatory and inhibitory molecules. The inhibitory receptors, also called immune checkpoints, regulate CTLs activation and effector function to maintain self-tolerance and limit bystander tissue damage as an indirect consequence of immune response against pathogenic invasion [5]. Targeting PD-1 and PD-L1, the immune checkpoint blockade agents could reactivate cytotoxic T cells to eliminate tumor cells.

This review will introduce PD-1, PD-L1 as well as the signaling pathway. It will also highlight the clinical development and research progress of the anti-PD-1 and anti-PD-L1 mAbs for managing categories of cancers. Except that, we interpret the aptamer that may become the next generation of PD-1/PD-L1 inhibitors and update the progress of tolerance mechanisms on PD-1/PD-L1 antibodies.

## 2. PD-1 (Programmed Cell Death Protein 1) and PD-L1 (Programmed Death-ligand 1) Signaling Pathway

### 2.1. PD-1

The *Pd-1* gene, classified to the immunoglobulin gene superfamily was isolated by Ishida Y using the subtractive hybridization technique in 1992 [6]. Like other inhibitory co-receptors, PD-1 has been detected on T cells, Tregs, exhausted T cells, B cells, activated monocytes, dendritic cells (DCs), natural killer (NK) cells and natural killer T (NKT) cells [7,8,9]. The PD-1 molecule consists of an intracellular domain which has potential phosphorylation sites located with immune tyrosine-based inhibitory motif (ITIM) and immune receptor inhibitory tyrosine-based switch motif (ITSM) and an extracellular IgV domain. Consequently there is a hydrophobic transmembrane region bridging crossing the cytomembrane [8]. Early studies have shown that an activated switch motif (ITSM) is required for the inhibitory effect of PD-1 on active T cells [10]. Its ITIM and ITSM also bind to the inhibitory phosphatase SHP-2 [11].

### 2.2. PD-L1

Two ligands, programmed death ligand-1 (PD-L1, CD274 or B7-H1) and programmed death ligand-2 (PD-L2, CD273 or B7-DC) [12], share 37% sequence homology [13,14,15]. PD-L1 belongs to type I transmembrane protein which was composed of extracellular domains (IgV-like domain, IgC-like domain, signal sequence), transmembrane domain and intracellular domains. PD-L1 constitutively express upon antigen presenting cells, non-lymphoid organs and non-hematopoietic cells such as heart, lung, placenta and liver [8]. Widely expressed PD-L1 is involved in self-tolerance, such as protecting peripheral tissues from excess of inflammation and autoimmune pathologies [16].

PD-L1 was induced by various pro-inflammatory cytokines like IFN-γ (interferon-γ), TNF-α (tumor necrosis factor-α), VEGF (vascular endothelial growth factor), GM-CSF (granulocyte-macrophage colony-stimulating factor) and IL-10. Activated T helper cells were responsible for IFN-γ and TNF-α and tumor stromal cells produced VEGF and GM-CSF. Furthermore up regulated PD-L1 expression in tumor cells facilitate immune suppression in tumor microenvironment [16] which has been called “adaptive immune resistance” [17]. In human cholangiocytes, PD-L1 expression was induced by IFN-γ and the MicroRNA -513 which complementary to 3′-untranslated region of PD-L1 mRNA, also could regulate PD-L1 translation. In other words, the miRNA could mediate gene silencing in the cholangiocyte regulation which respond to IFN-γ [18]. While in human glioma, PD-L1 expression would be increased if the tumor suppressor phosphatase and tensin homolog (PTEN), were dysfunctional and the phosphatidylinositol-3-OH kinase (PI(3)K) pathway were in turn activated [19]. In contrast, PI3K could increase translation of PD-L1 mRNA and cause the high expression of PD-L1 protein [20]. IFN-γ inducible PD-L1 expression was also dependent on NF-κB [21]. Except for binding PD-1, PD-L1 also binds to CD80 to deliver an inhibitory signal inducing T cell tolerance [22].

### 2.3. PD-1 and PD-L1 Pathway

Under normal physiological conditions, PD-1 which acts as immune checkpoint could interact with its two ligands, PD-L1 and PD-L2, and plays a very important part in lowering the immune system though suppression of T-cells function, upregulating regulatory T cells (Treg), which in turn reduces autoimmunity and promotes self-tolerance [23,24]. After binding of PD-L1 or PD-L2, the recruitment of tyrosine phosphatases will begin and then generates an inhibitory signal blocking the downstream effects of PI3K/Akt pathway leading to cell cycle arrest and suppressed T-cell activation [10,25].

Varieties of cancer cells have been detected through PD-L1 expression including melanoma, multiple myeloma, leukemia, glioblastoma as well as gastric, renal cell, bladder, hepatocellular, cutaneous, breast and NSCLC (Non-Small Cell Lung Cancer) [26,27,28,29,30,31,32,33], whereas PD-1 have been highly detected on tumor-infiltrating lymphocytes (TILs) [34,35]. Apart from PD-L1 displaying on camera solid tumors, PD-L2 (as well as PD-L1) is conservatively expressed in a few subsets of B cell lymphomas [36]. When cancer cells are attacked by the immune system, they start to overexpress PD-L1 and PD-L2, for impacting T-cells efficiency. After that, T cells will be suppressed successfully, leading to immune escape [37].

In diverse forms of tumor microenvironment, T-cell viability suppressed by PD-1 and its ligand PD-L1 though various mechanisms. It has been demonstrated that overexpression of PD-L1 on tumor associated macrophages, DCs, MDSCs and tumor cells positively correlated with the exhaustion of TILs in the tumor [38]. PD-L1 could induce Treg cell (iTreg cell) development by the down-regulating of phospho-Akt, mTOR, S6 and ERK2 accompanied with PTEN up-regulating. These signaling molecules play a critical role in iTreg cell development. As a consequence, PD-L1 will inhibit T cell activation though the formation and holding of iTreg cells [9]. On the other hand, earlier researcher Dong H and colleagues have proven that PD-1 inhibits PI3K activation inducing cell death in activated T cells resulting in the down regulation of anti-apoptotic protein Bcl-xL [39]. What is more, PD-1 could also inhibit the T-cell receptor delivering downstream, which is required for production growth stimulatory IL-2, resulting in cell cycle arrest and blocking T cell proliferation [40].

## 3. Programmed Death 1 Inhibitors

In 1996, Jim Allison’s group found that anti-CTLA-4 could boost anti-tumor response of T cell, which proved the immune checkpoint blocking in tumor therapy for the first time [2]. Through 10 years’ tough clinical research, the FDA finally approved the ipilimumab in 2011. Except for the different efficacy profile, the immune-related adverse events (irAEs), which were studied in-depth and accepted by the FDA, were regarded as an example of a new immunotherapy safety [41] which drove the clinical trials of the PD1 and PD-L1 antibody. Because of the lessons learned from ipilimumab and tremelimumab, nivolumab and pembrolizumab was marketed without difficulty in 2014.

### 3.1. Nivolumab

Nivolumab (BMS-936558 or MDX1106b) is a human IgG4 antibody against PD-1, lacking detectable antibody-dependent cellular toxicity (ADCC). It is manufactured by Bristol-Myers Squibb Company Princeton and has been approved by the FDA for the use of unresectable or metastatic melanoma, metastatic NSCLC and advanced renal cell carcinoma (Table 1).

#### 3.1.1. Unresectable or Metastatic Melanoma

##### Nivolumab as a Single Agent for Melanoma

A multi-center, double-blind, randomized (1:1) clinic trial data of patients with BRAF V600 wild-type unresectable or metastatic melanoma is shown in the Table 2. A much higher overall survival rate of 1 year OS in the nivolumab group has been reached, 72.9% comparing to 42.1% in dacarbazine. Progression-Free-Survival (PFS) was also statistically significant in contrast to dacarbazin [42].

##### Nivolumab in Combination with Ipilimumab for Melanoma

A total of 109 randomized (2:1) patients, previously untreated with unresectable or metastatic melanoma participated the double-blind clinic trial. They received either ipilimumab or combined with nivolumab. In all 43 patients, 21% (9) had a response within the duration time from three to seven months. Unfortunately at end of trail, some progressed, others died or received other therapy. Among the remaining thirty four patients, fourteen had at least six months and less than nine months in duration of ongoing responses while the remaining 20 patients had a long duration of ongoing responses of more than nine months [43]. In contrast to single-agent ipilimumab, the combination group had a statistically significant increased ORR (overall response rate) in this study (Table 3).

#### 3.1.2. Metastatic Non-Small Cell Lung Cancer

##### Second-Line Treatment of Metastatic Squamous NSCLC

The clinic trial named CheckMate017 phase 3, studied 272 patients suffering from metastatic squamous NSCLC and followed their progression during or after platinum doublet-based chemotherapy. The median overall survival rate was 9.2 months with nivolumab versus 6.0 months with docetaxel. The overall survival rate was 42% with nivolumab versus 24% with docetaxel at 1 year (Table 4).

##### Second-Line Treatment of Metastatic Non-Squamous NSCLC (Non-Small Cell Lung Cancer)

Nivolumab had been expanded to NSCLC by the FDA on October 9, 2015 based on the CheckMate 057 trial [44] with 582 patients enrolled. Nivolumab improved OS from 9.4 months (docetaxel 95% CI: 8.0–10.7 months) to 12.2 months (nivolumab 95% CI: 9.7–15.0 months) with a hazard ratio (HR) of 0.73 (*p* = 0.0015) (Table 5).

#### 3.1.3. Renal Cell Carcinoma

A phase 3 study in 821 renal-cell carcinoma patients who have experienced previous treatment was completed in comparing nivolumab(OS 25.0 months) with everolimus(OS 19.6 months). OS benefit was significantly confirmed even if the expression of PD-L1 could not be detected. The hazard ratio for death was 0.73 (98.5% CI, 0.57 to 0.93; *p* = 0.002) when nivolumab was combined with everolimus [45]. Table 6 displays further information.

#### 3.1.4. Adverse Reactions

After the immune checkpoints were blocked, the balance between the autoimmunity and immune tolerance were broken as well. Newly generated dysimmune toxicities created immune-meditated adverse reactions (IMARs) caused by the new immunotherapy. For instance, immune-mediated pneumonitis, colitis, hepatitis, endocrinopathies, rash, encephalitis and other immune-mediated adverse reactions were observed as IMARs. Immune-mediated pneumonitis, colitis, hepatitis, nephritis and renal dysfunction, meant patients required the use of corticosteroids and had no clear alternative etiology, which can occur with nivolumab treatment. Immune-mediated edocrinopathies and rash mainly occurred in combination with ipilimumab. A total of 8,490 patients received nivolumab as a single agent or in combination with ipilimumab in all clinical trials. Fortunately, less than 1.0% of them were confirm as having encephalitis. As well as less than 1.0% of patients were regarded as having severe in fusion when using nivolumab as a single-agent. In only one patient (0.3%) did fatal limbic encephalitis occur, after receiving nivolumab after 7.2 months of exposure. Others were administered with corticosteroids. In addition, the fetus could be harmed when pregnant woman received nivolumab treatment, based on data from animal studies.

In clinical trials, the most common adverse reactions experienced (≥20%) in melanoma were fatigue, musculoskeletal pain, rash and pruritus when nivolumab acted as a single-agent. Patient symptoms when Nivolumab was used in combination with ipilimumab were rash, pruritus, headache, vomiting and colitis.

The most common adverse reactions (≥20%) in patients with metastatic NSCLC were fatigue, musculoskeletal pain, decreased appetite, cough and constipation, based on clinical trials experience.

Just like other therapeutic proteins, nivolumab also has the potential of immunogenicity. 73 patients (11.4%) was tested positive for anti-nivolumab antibodies because of the treatment and using an electrochemiluminescent (ECL) assay in all 639 patients. In five patients (0.8%), anti-nivolumab neutralizing antibodies were detected as well. In combinational therapy with ipilimumab, 23 patients were tested positive for treatment-arising anti-nivolumab antibodies though ECL assay and neutralizing antibodies, anti-nivolumab were inspected at one patient.

#### 3.1.5. Recruiting Clinical Trials of Nivolumab

**Figure 1:**
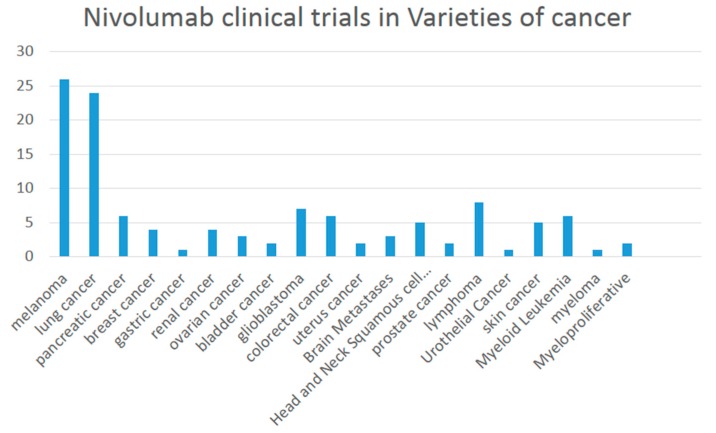
Nivolumab clinical trials in a variety of cancers.

There are 121 studies found in ClinicalTrials.gov searching for “Nivolumab | Recruiting”. A variety of melanoma are involved including lung cancer, breast cancer, bladder cancer and renal cancer (Figure 1) when combined or compared with multiple medicines such as Ipilimumab, Pembrolizumab, Dabrafenib, Trametinib, Placebo, Omaveloxolone Capsules, sunitinib, BMS-936558 (MDX1106-04), Fotemustine and HyperAcute^®^-Melanoma (HAM) Immunotherapy.

### 3.2. Pembrolizumab

Pembrolizumab (MK-3475, lambrolizumab, KEYTRUDA) is an highly specific anti-PD-1 humanized IgG4 κ isotype antibody that contains a mutation at C228P designed to prevent Fc-mediated cytotoxicity. It can disrupt the engagement of PD-1 and PD-L1, resulting in tumor recognition by cytotoxic T cells.

It is approved by the FDA for the treatment of the patients suffering with unresectable or metastatic melanoma and patients with metastatic NSCLC. Accordingly, the PD-L1 expression level has to be detected in determining whether the patients receive pembrolizumab or not. Otherwise, their disease continued to progress on or after chemotherapy platinum. In the case of patients with EGFR or ALK, genomic aberrations had disease progression on other prior FDA-approved therapy before receiving pembrolizumab.

#### 3.2.1. Pembrolizumab in Melanoma

##### Ipilimumab-Naive Melanoma

The safety and efficacy of pembrolizumab were well demonstrated in Ipilimumab-Naive Melanoma. The affirmed 6-month PFS rates were 47.3% for the patients receiving pembrolizumab every 2 weeks contrasting with 46.4% for every 3 weeks and 26.5% for ipilimumab, with 12-month survival rates: 74.1%, 68.4% and 58.2%, respectively. The improving response rate, regardless of the interval of pembrolizumab being administered; whether every 2 weeks (33.7%) or every 3 weeks (32.9%), as compared with ipilimumab (11.9%). The response rates were ongoing in 89.4%, 96.7%, and 87.9% of patients, respectively. All the data supports the efficiencies were similar in two pembrolizumab patients groups. The adverse events rates referring to treatment of grade 3 to 5 severity were lower in the pembrolizuma patients (13.3% and 10.1%) than in the ipilimumab patients (19.9%) [46,47] (Table 7). These clinical trials suggests there are statistically significant improvements in OS and PFS for patients receiving pembrolizumab in Ipilimumab-Naive Melanoma.

##### Ipilimumab-Refractory Melanoma

A safety and efficacy phase II trial of pembrolizumab were evaluated in Ipilimumab-Refractory Melanoma. In this trial by active comparator arms, the pembrolizumab group had significantly improved PFS and ORR but not OS (although OS data are immature), when compared with BRAF/MEK inhibiting chemotherapy in ipilimumab-refractory patients with BRAF-mutation positive [47,48,49,50,51] (Table 8). After an analysis of 220 deaths, there was no statistically significant difference between pembrolizumab and chemotherapy, regardless of the dosage of pembrolizumabpembrolizumab, 2 mg/kg or 10 mg/kg (Table 8).

#### 3.2.2. Pembrolizumab in Non-Small Cell Lung Cancer

Altogether, 280 patients were involved in a multi-center, open-label multi-cohort, activity-estimating study. The sub-group defining was retrospectively analyzed using an analytically validated assay for PD-L1 expression TPS (tumor proportion score). Of a total of 280 patients, 61 were defined as highly expressed for PD-L1 with partial response, consequently the confirmed ORR reached to 41% (Table 9). In 25 ORR patients, 21 (84%) had duration response, as well as 11 patients (44%) who had ongoing response to ≥6 months [52].

#### 3.2.3. Adverse Reactions

In clinical trials research, the adverse reactions reported in ≥20% of patients were fatigue, pruritus, rash, constipation, diarrhea, nausea with decreased appetite and fatigue, dyspnea and cough in NSCLC.

In total, 2117 patients with melanoma, 1567 NSCLC 550 patients, pembrolizumab caused some immune-mediated adverse reaction such as immune-mediated pneumonitis, colitis, hepatitis, endocrinopathies, nephritis and renal dysfunction. The exact percentage is shown in (Table 10).

Similar to other therapeutic proteins, the immunogenicity risk for pembrolizumab was observed in clinical studies. The anti-pembrolizumab antibody were detected in 1 (0.3%) of 392 patients which were verified positively in the neutralizing assay.

#### 3.2.4. Clinical Trials on Recruiting

**Figure 2:**
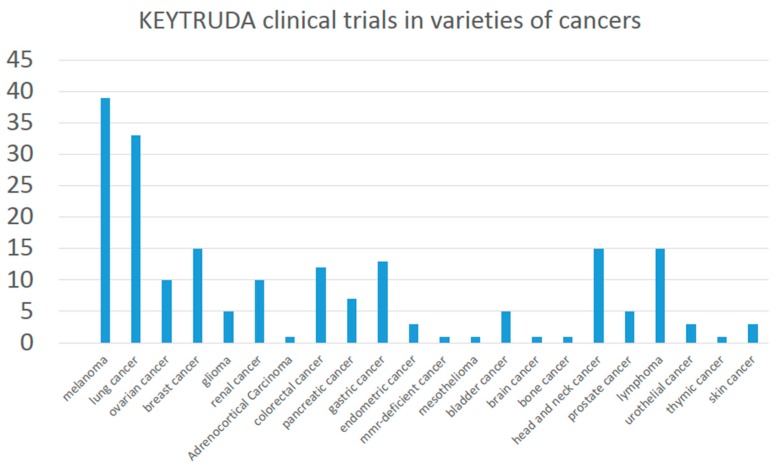
Pembrolizumab clinical trials in varieties of cancer.

There were 178 studies in the ClinicalTrials.gov with recruited participants distributed throughout the world. The 178 clinical trials used pembrolizumab in many varieties of cancer, including melanoma, lung, ovarian, breast, glioma, renal, adrenocortical carcinoma, colorectal, pancreatic, gastric, endometrial, mesothelioma, bladder cancer and so on (Figure 2).

### 3.3. Pidilizumab

Pidilizumab targeting PD-1 is derived from BAT, a the mouse monoclonal antibody, and it was humanized to IgG 1κ [53]. In preclinical studies, CT-011 and BAT had successfully suppressed the tumor growth within melanoma, lymphoma, lung, colon and breast tumors and furthermore it extended the survival of tumor-bearing mice, both NK and T cell involved [54,55,56,57]. The Phase I study has affirmed the safety and pharmacokinetic of pidilizumab in advanced ematologic malignancies. Fortunately, there was no observed treatment or infusion-related serious adverse events [58]. Pharmacokinetic analyses show the Cmax (maximum concentration) and the AUC (area under the curve) of CT-011 in serum increasing dose with a median of t1/2 from 217 to 410 h. The peripheral blood CD4+ lymphocytes rose unremittingly until the 21st. day after CT-011 treatment.

Researchers designed a single group, open-label phase 2 trial, for assessing the safety and activity of the combination between Pidilizumab and Rituximab in relapsed Follicular Lymphoma patients. It was demonstrated that no autoimmune or therapy-related grade 3/4 toxicities were observed. Anemia (14 patients) and fatigue (13 patients) were the most frequent grade 1 adverse symptoms and 5 patients were defined as having respiratory infection with a grade 2 adverse event. Overall response rates were 66% (19/29), 15 being complete response rates. In total, 25/29 (86%) of the patients had tumor regression within 18.8 months (95% CI: 14.7 months to not reach) of median progression-free survival. Nineteen responders had 20.2 months (95% CI: 13.9 months to not reached) of median response duration [53].

The clinical activity of PD-1 blockade was confirmed in diffused large B-cell lymphoma (DLBCL) using a phase II trial (NCT00532259). After autologous hematopoietic stem-cell transplantation for DLBCL, the overall response rate of pidilizumab treatment has been reported as reaching 51% which presents a promising strategy of PD-1 blockade therapy. It is demonstrated in DLBCL for the first time. A total of 613 adverse events took place in 69 (96%) patients and 135 were regarded as treatment-related. Neutropenia (19%) and thrombocytopenia (8%) became the most common grade 3 to 4 adverse events but the neutropenia could be managed by growth factor treatment and remaining in asymptomatic. Sadly, one patient died due to herpes zoster infection after the third dose of pidilizumab, but is not related to this study treatment. At least 32% (23/72) of patients suffered one serious adverse event each with three undergoing a related serious adverse event. The evidence of significant autoimmune toxicity, infusion reactions and treatment related mortality have not yet been found [59].

Pidilizumab was studied in another two Phase 2 clinic trials on aggressive and indolent lymphomas which appeared in clinical activity of PD-1/PD-L1 positive lymphocytes. Then Michael B etc. initiated a large Phase 2 study to assess the safety and efficacy of pidilizumab in metastatic melanoma (MM) patients. It resulted in a substantial 12 month survival rate in heavily pretreated patients and was very well tolerated. It appears comparable to other anti- PD-1 MAbs in 12 months survival [60].

There were also two clinical trials but these were suspended due to a Pidilizumab (CT-011) licensing transfer and Pharmaceutical Companies decision. Two further clinical trials with Chronic Hepatitis C Genotype and Hepatocellular Carcinoma were terminated. The study of PD-1 blockading in combination with DC/AML vaccine and subsequent chemotherapy to induce remission is being sought. A Stage III-IV diffuse large B-cell lymphoma trial is requesting volunteer patients as well.

### 3.4. AMP-224

The curative effects of nivolumab and pembrolizumab are very encouraging [61]. However, targeting the PD-1 has the potential to prevent differentiated development [62,63]. Amplimmune and GlaxoSmithKline are assessing the safety and efficiency of a new arising PD-1 targeting agent, AMP-224. It is a fusion protein consist of the extracellular domain of PD-L2 and the Fc region of human IgG [64]. Contrasting to nivolumab and pembrolizumab, AMP-224 does not just perform as a blockading agent. It is hypothesized that AMP-224 could deplete PD-1 highly expressed T-cells, which referred to as exhausted effector cells, ADCC or CDC. Following restoration of the T-cell cohort with normal function may reestablish immune ability [2,3]. AMP-224 was well-tolerated up to its maximum administered dose of 30 mg/kg, with manageable infusion reactions in the majority of patients. The trial is ongoing, including monitoring for clinical activity [65].

Preclinical studies utilizing a murine AMP-224 in syngeneic murine tumor models show anti-tumor activity as a single agent which could be enhanced after the combination of low dose cyclophosphamide (unpublished data Amplimmune, Inc, Gaithersburg, MD, USA).

A pilot research of AMP-224 combined with stereotactic body radiation therapy (SBRT) in patients with metastatic colorectal cancer was reported at the 2015 Gastrointestinal Cancers Symposium. A few other preclinical studies have reported an increase in peripheral anti-tumor immune activity, consequently with radiation for “abscopal effect”. The PD-L1 expression of tumor cells could also been induced by radiation, so the aim of this study is to assess whether the radiation therapy enhanced anti-tumor immunity of anti-PD-1 therapy (with AMP-224) or not. However, the clinical trial information is not yet complete [66].

### 3.5. MEDI0680

MEDI0680 (AMP-514) is a humanized IgG monoclonal antibody targeting human PD-1. It could also improve the cytotoxicity of T cells though inducing the internalization of PD-1 [67,68]. There are three ongoing clinic trails: NCT02271945 is a Phase 1b/2 open-label study to evaluate the safety/efficacy of MEDI-551 + AMP-514 in participants with relapsed or refractory aggressive B-cell lymphomas who have failed 1–2 prior lines of therapy; NCT02013804 is a dose-escalation study to test the safety, tolerability, PK, immunogenicity and anti-tumor activity in adult patients bearing solid tumors; NCT02118337 is a combinational trail to assess the safety and tolerability of AMP-514, with MEDI4736 (Anti-PD-L1 Antibody).

### 3.6. REGN2810

REGN2810 is a fully human hinge-stabilized IgG4 monoclonal Ab that binds to the extracellular domain of human PD-1 with high affinity and specificity inhibiting interaction of PD-1 with its ligands [69]. Elena Burova et al. generated a mouse with human PD-1 gene knock-in allowing direct testing of our anti-human PD-1 Ab. Human PD-1 knock-in mice express a hybrid protein containing the extracellular portion of human PD-1 with transmembrane and intracellular domains of mouse PD-1. We demonstrated functional PD-1/PD-L1 signaling and immune responses in this model and confirmed REGN2810 binding to hybrid PD-1 receptor on mouse T cells in vivo, following REGN2810 injections. Prophylactic and therapeutic treatments of subcutaneous syngeneic tumors with REGN2810 in human PD-1 knock-in mice resulted in a dose-dependent suppression of tumor growth [69,70].

Three phase I clinic trials are commencing at clinicaltrials.gov. NCT02383212 is an open-label, multi-center, ascending-dose escalation study of REGN2810, alone and in combination with other anti-cancer therapies in patients with advanced malignancies on recruiting. NCT02651662 is calling for participants in patients with lymphoma. It is an open-label, multi-center, dose escalation study of REGN2810 as single-agent. NCT02520245 has been designed to collect long-term follow-up information for patients who received REGN2810 in other clinical studies and to allow re-treatment for eligible patients not yet recruiting.

### 3.7. PDR001

PDR001 is a high-affinity, ligand-blocking, humanized anti-PD-1 IgG4 antibody that blocks the binding of PD-L1 and PD-L2 to PD-1. As a signal agent, NCT02678260, NCT02605967 and NCT02404441 are on phase I/II. NCT02404441 and is the “first-in-human” study of PDR001 to characterize the safety, tolerability, pharmacokinetics (PK), pharmacodynamics (PD) and anti-tumor activity of PDR001 administered *i.v.* as a single agent, to adult patients with solid tumors. The purpose of NCT02678260 is to characterize the safety, tolerability, Pharmacokinetics (PK) and anti-tumor activity of PDR001 administered intravenous as a single agent to Japanese patients. The randomized controlled of NCT02605967 Phase II study is to assess the efficacy of PDR001 versus investigator's choice of chemotherapy in patients with advanced NPC. Another two agents (NCT02608268 and NCT02460224) were in combination with MBG453 or LAG525.

### 3.8. PD-1 Antibody in China

China has overtaken India becoming the largest biosimilar discovering country from Cortellis Competitive Intelligence, according to Thomson Reuters report in 2015. It mirror the investment environment’s expectation to Chinese biosimilars. There is no doubt that the development of biosimilar will make up for the massive unsatisfied clinic demand in diabetes, tumor and immunological diseases.

Nivolumab and pembrolizumab have submitted an application for clinic trials, but most are in assessing, except for a clinic trial of nivolumab (JXSL1300032). As expected, there are many ongoing PD-1 antibodies discoveries in China. According to incomplete statistics, two have reached clinical trials and a further two are in assessment (Table 11).

## 4. Programmed Death Ligand 1 Inhibitors

PD-L1 constitutively express upon antigen presenting cells, non-lymphoid organs and non-hematopoietic cells such as heart, lung, placenta and liver [8]. Early studies, reported by many groups respectively, also have shown that PD-L1 is frequently expressed on human cancer cells which significantly correlate with the poor prognosis in various kinds of tumor (e.g., renal, gastric, urothelium, ovarian and melanoma) [71]. Thus using the anti-PD-L1 antibody could kill the tumor cell or block the PD-1/PD-L1 signal pathway to reactivate CTLs. The first therapeutic anti-PD-L1 antibody has been approved by the FDA on 18 May 2016 and immediately made headlines in the media.

### 4.1. BMS-936559

BMS-936559 is a PD-L1 specific, fully human, high-affinity, IgG4 (S228P) monoclonal antibody that inhibits the interaction of PD-L1 to PD-1 or CD80 [72]. NCT00729664 is a phase I clinic trial commenced in 2008 with the report was published in 2012. A total of 207 patients were included, with NSCLC (75), melanoma (55), colorectal cancer (18), renal-cell cancer (17), ovarian cancer (17), pancreatic cancer (14), gastric cancer (7) and breast cancer (4). The duration of therapy ranged from 2 to 111 weeks, with a median of 12 weeks. The researchers considered 9% of patients treatment related, Grade 3 or 4 toxic effects. An objective response, both complete and partial, was counted in 9/52 patients with melanoma; 2/17 patents with renal-cell cancer, 5/49 patients with NSCLC and 1/17 patient with ovarian cancer who were evaluated to have an objective response [72]. Of 8/16 patients, the response lasted for more than one year and at least a further year of follow-up. Another NCT02028403 trial was completed in safety and immune response of BMS-936559 of HIV-infected patients followed up with antiretroviral therapy. The purpose of NCT02576457 was to determine whether BMS-936559 is safe and has the desired pharmacologic activity in patients who have severe sepsis and are being sought for trial. However, there are two clinic trials (NCT01455103 and NCT01452334) that were withdrawn prior to enrollment.

### 4.2. Avelumab

In November 2014, Merck KGaA, Darmstadt, Germany and Pfizer proclaimed the avelumab would be co-developed and co-commercialized within a strategic alliance. Avelumab (MSB0010718C) is an investigational product consist of fully human anti-PD-L1 IgG1 monoclonal antibody. Though the blockading of PD-L1, avelumab is regarded as a function in the reactivation of T-cells and may induce ADCC with native Fc-region [73]. Altogether 16 clinical trials in three phases were active with 12 recruiting rounds (Table 12).

### 4.3. MEDI4736

MEDI4736 is a human IgG1 κ monoclonal antibody with high affinity and specificity to PD-L1, which also been engineered to prevent ADCC. It significantly suppresses the growth of human tumors in a new xenograft model with co-implanted human T cells and is entirely dependent on the existence of human T cells [74,75]. Antibodies of the IgG1 isotype antibody could trigger cytotoxic effect or functions, such as ADCC activity and CDC (complement-dependent cytotoxicity) [76]. Fc (the fragment crystallizable) domain of the antibody molecule having a triple mutation of the IgG1 heavy chain, for reducing the interaction with the complement component C1q and the Fcγ receptors [74,76,77,78]. The absence of ADCC and CDC effector functions has been confirmed using cell-based functional assays [78,79]. Many anticipated clinic trials are ongoing (Table 13).

### 4.4. MPDL3280A

MPDL3280A (also known as Atezolizumab, Tecentriq) is a phage-derived human IgG1 monoclonal antibody targeting PD-L1 and has shown promising anti-tumor activity [80]. Similarly to MEDI4736, MPDL3280A was also engineered with a mutation in the Fc domain (298 Asn to Ala) [81]. In a preclinical study in monkeys, MPDL3280A has shown pleasing results in patients with locally advanced or metastatic tumors [82,83]. Furthermore, on 18 May 2016, it became the first PD-L1 inhibitor post FDA approval of the treatment of urothelial carcinoma, the most common type of bladder cancer. The other 51 clinic trials were recruited around the world (Table 14).

## 5. Prospects

To date, three therapeutic antibodies targeting the PD-1/PD-L1 signal pathway have been approved by the FDA in the use of metastatic melanoma, NSCLC, renal cell cancer or urothelial carcinoma, with several others in clinical trials in preparation for release to the open market (Table 15) [84]. It is predicted that some will be approved.

Varied response towards immunotherapy has resulted from the different immune backgrounds of patients. The immune background is dependent on many substances with the immunogenicity a person has in their immune system being determined in the womb. However, the gut microbiota plays a most important role in shaping the systemic immune responses [85,86,87]. Ayelet Sivan demonstrate the commensal Bifidobacterium could promote antitumor immunity and facilitate anti-PD-L1 activity in cancer [88]. So, it is possible to manipulate microbiota to modulate cancer immunotherapy. On the other hand, it has been a long journey for Ipilimumab due to the low responsible rate compared to small molecular drugs or other therapeutic antibodies. Similarly, the responsible rate of PD-1/PD-L1 antibodies were still low as immunotherapeutic antibodies. Most researchers view that the different performances in a variety of people are due to the individual immune system and tumor driven mutation. However, which gene has been a mystery, until recently. A researcher from University of California, Los Angeles found that highly mutational loads co-related with optimistic survival and the responding patients’ tumors are abundant with mutations in BRCA2. Similarly, mitogen-activated protein kinase (MAPK) inhibited therapy induces similar characteristics in melanoma, which indicate that non-genomic MAPK inhibitor resistance has the cross-resistance within anti-PD-1 therapy [89].

To address the immune-related adverse effects of mAbs and get more penetration in solid tumors, some peptides based on immune checkpoint blockers were discovered. A therapeutic peptide targeting PD-1/PD-L1 signal pathway for cancer immunotherapy, AUNP-12 (AUR-12/Aurigene-012) was co-developed by Aurigene Discovery Technologies and Pierre Fabre Laboratories and is currently undegoing preclinical study [84]. The sequence is still secret. Although it has shown valid antitumor activity, the druggability pharmacokinetic profile was too short. Some hydrolysis-resistant D-peptides were discovered as PD-L1 antagonists developed by using mirror-image phage display [90]. The highest affinity of Kd = 0.51 μM has shown inhibited tumor growth and prolonged animal survival.

Otherwise, there was one patent relating to small drug-like inhibitors. It was filed by Bristol-Myers Squibb in 2015 WO 2015/034820 A1 (priority to US 61/873,398). The compound and its ramification could inhibit the interactions between PD-1 and PD-L1 having IC50 values between 0.006 and 0.10 mM. It should be noted that Curis, Inc. (Nasdaq:CRIS, Lexington, MA, USA) announced the FDA has accepted the company’s Investigational New Drug (IND) application for CA-170 at 1 June 2016. CA-170 is the first orally available small molecule targeting and inhibiting the immune checkpoints, PD-L1 and V-domain Immunoglobulin Suppressor of T-cell Activation (VISTA).

Since the regulatory approval of ipilimumab in 2011, immuno-oncology has not only grown rapidly but also inspired the entire pharmaceutical industry. Dozens of new technologies and investments in immune-oncology have soared overnight, especially since *Science* magazine in 2013 reported cancer immunotherapy as the breakthrough of the year [91]. Between 2014 and 2015, immune-oncology technologies and business deals led to extreme valuations and the market is expected to reach US $35 billion by 2023 [92]. Axel Hoos also reviewed or forecast three generations of immuno-oncology drugs. Generation 1 encompasses the initiating antibodies of the immune-oncology era, such as ipilimumab and sipuleucel‑T (autologous dendritic cell therapy developed by Dendreon), which were approved respectively in 2011 and 2010 [93,94]; and then immune-oncology drugs rapidly develop to next generation agents against new targets and new emerging mechanisms, which are represented by the PD-1 and PD-L1 antibodies. A bi‑specific T cell adaptor (BITE), blinatumomab (Amgen) targeting CD19 + B cell malignancies and T cell, was also approved in 2015 [95,96,97]. Generation 3 will be various immune-oncology modalities combined with adaptive immunity and innate immunity. In other words, establishing the best combination of therapies and drugs is under development. Further development of PD-1/PD-L1 pathway inhibitors are expected to be a powerful weapon in the fight against cancers.

We expected more PD-1 (or PD-L1) inhibitors to show excellent results at immune checkpoints in reactivating the adaptive or innate immunity for defeating cancers. However, antibody drugs have some insurmountable disadvantages, such as high immunogenicity [98], high cost, low stability and low production [99]. Undoubtedly, the marketed two PD-1 antibodies have been reported with anti-antibodies which weaken the therapeutic effect. The anti-antibody of nivolumab (OPDIVO) has reached up to 12.6% (67/532) and so a new solution may be required. The aptamer with minimal immunogenicity [100], low cost [101], high production [101] and high stability [99] may became the key to solving the puzzle. More and more anti-tumor drugs will be investigated until we overcome cancer.

## Figures and Tables

**Table 1:** The Indication of Nivolumab.

Cancers	Single Agent	Combination with Ipilimumab
Melanoma	BRAF V600 wild-type unresectable or metastatic melanoma	BRAF V600 wild-type unresectable or metastatic melanoma
	Unresectable or metastatic, BRAF V600 mutation-positive melanoma and disease progression following ipilimumab and a BRAF inhibitor	
NSCLC	Metastatic non-small cell lung cancer in patients with progression on or after platinum-based chemotherapy	
Renal cancer	Advanced renal cell carcinoma in patients who have received prior antiangiogenic therapy	

**Table 2:** Efficacy Results of Nivolumab as a Single Agent for Melanoma.

Efficacy	Nivolumab (*n* = 210)	Dacarbazine (*n* = 208)
**Overall Survival**		
Events (%)	50 (24)	96 (46)
Median, months (95% CI)	Not Reached	10.8 (9.3, 12.1)
Hazard ratio (95% CI)	0.42 (0.30, 0.60)	
*p*-Value	<0.0001 ^a^	
**Progression-Free Survival**		
Events (%)	108 (51)	163 (78)
Median, months (95% CI)	5.1 (3.5, 10.8)	2.2 (2.1, 2.4)
Hazard ratio (95% CI)	0.43 (0.34, 0.56)	
*p*-Value	<0.0001 ^a^	
Objective Response Rate (95% CI)	34% (28, 41)	9% (5, 13)
Complete response rate	4%	1%
Partial response rate	30%	8%

^a^ *p*-Value is compared with the allocated alpha of 0.0021 for this interim analysis.

**Table 3:** Efficacy Results of Nivolumab in Combination with Ipilimumab for Melanoma.

Endpoint	Nivolumab Plus Ipilimumab ^a^ (*n* = 72)	Ipilimumab (*n* = 37)
**Objective Response Rate (95% CI)**	60% (48, 71)	11% (3, 25)
Difference in ORR (95% CI)	49 (31, 61)	
*p*-Value	<0.001	
CR (%)	17%	0
PR (%)	43%	11%
**Progression-Free Survival**		
Number of events	27	23
Median PFS (months) (95% CI)	8.9 (7.0, NA)	4.7 (2.8, 5.3)
Hazard ratio (95% CI)	0.40 (0.22, 0.71)	
*p*-Value	<0.002	

NA: not available; ^a^ Nivolumab 1 mg/kg in combination with ipilimumab 3 mg/kg every 3 weeks for 4 doses, followed by nivolumab 3 mg/kg as a single agent every 2 weeks until disease progression or unacceptable toxicity.

**Table 4:** Efficacy Results of Nivolumab as Second-Line Treatment of Metastatic Squamous NSCLC.

Efficacy	Nivolumab (*n* = 135)	Docetaxel (*n* = 137)
Prespecified Interim Analysis		
Events (%)	86 (64%)	113 (82%)
Median survival in months (95% CI)	9.2 (7.3, 13.3)	6.0 (5.1, 7.3)
*p*-Value ^a^	0.00025	
Hazard ratio (95% CI) ^b^	0.59 (0.44, 0.79)	

^a^ *p*-Value is derived from a log-rank test stratified by region and prior to paclitaxel use; the corresponding O’Brien-Fleming efficacy boundary significance level is 0.0315; ^b^ Derived from a stratified proportional hazards model.

**Table 5:** Efficacy Results of Nivolumab as Second-Line Treatment of Metastatic Non-Squamous NSCLC (non-small cell lung cancer).

Efficacy	Nivolumab (*n* = 292)	Docetaxel (*n* = 290)
**Overall Survival**		
Deaths (%)	190 (65%)	223 (77%)
Median (months) (95% CI)	12.2 (9.7, 15.0)	9.4 (8.0, 10.7)
*p*-Value ^a,b^	0.0015	
Hazard ratio (95% CI) ^c^	0.73 (0.60, 0.89)	
**Objective Response Rate**	56 (19%)	36 (12%)
(95% CI)	(15, 24)	(9, 17)
*p*-Value ^d^	0.02	
Complete response	4 (1.4%)	1 (0.3%)
Partial response	52 (18%)	35 (12%)
Median Duration of response (months)	17	6
**Progression-Free Survival**		
Disease progression or death (%)	234 (80%)	245 (84%)
Median (months)	2.3	4.2
*p*-Value ^a^	0.39	
Hazard ratio (95% CI) ^c^	0.92 (0.77, 1.11)	

^a^ Based on stratified log-rank test; ^b^ p-value is compared with .0408 of the allocated alpha for this interim analysis; ^c^ Based on a stratified proportional hazards model; ^d^ Based on the stratified Cochran-Mantel-Haenszel test.

**Table 6:** Efficacy Results of Nivolumab for Renal Cell Cancers.

Efficacy	Nivolumab (*n* = 410)	Everolimus (*n* = 411)
**Overall Survival**		
Events (%)	183 (45)	215 (52)
Median survival in months (95% CI)	25.0 (21.7, NE)	19.6 (17.6, 23.1)
Hazard ratio (95% CI) ^a^	0.73a (0.60, 0.89)	
*p*-Value ^b^	0.0018 ^b^	
**Confirmed Objective Response Rate (95% CI)**	21.5% (17.6, 25.8)	3.9% (2.2, 6.2)
Median duration of response in months (95% CI)	23.0 (12.0, NE)	13.7 (8.3, 21.9)
Median time to onset of confirmed response in months (min, max)	3.0 (1.4, 13.0)	3.7 (1.5, 11.2)

NE; not estimatble; ^a^ Hazard ratio is obtained from a Cox proportional-hazards model stratified by MSKCC risk group, number of prior anti-angiogenic therapies and region with treatment as the sole covariate; ^b^ *p*-value is obtained from a two-sided log-rank test stratified by MSKCC risk group, number of prior antiangiogenic therapies and region. The corresponding O’Brien-Fleming efficacy boundary significance level is 0.0148.

**Table 7:** The Clinical Study of Pembrolizumab for Ipilimumab-Naive Melanoma (Trial 6).

Efficacy	Pembrolizumab 10 mg/kg Every 3 Weeks *n* = 277	Pembrolizumab 10 mg/kg Every 2 Weeks *n* = 279	Ipilimumab 3 mg/kg Every 3 Weeks *n* = 278
**OS**			
Death (%)	92 (33%)	85 (30%)	112 (40%)
Hazard ratio * (95% CI)	0.69 (0.52, 0.90)	0.63 (0.47, 0.83)	–
*p*-Value (stratified log-rank)	0.004	<0.001	–
**PFS by BICR**			
Events (%)	157 (57%)	157 (56%)	188 (68%)
Median in months (95% CI)	4.1 (2.9, 6.9)	5.5 (3.4, 6.9)	2.8 (2.8, 2.9)
Hazard ratio * (95% CI)	0.58 (0.47, 0.72)	0.58 (0.46, 0.72)	–
*p*-Value (stratified log-rank)	<0.001	<0.001	–
**Best Overall Response by BICR**			
ORR % (95% CI)	33% (27, 39)	34% (28, 40)	12% (8, 16)
Complete response %	6%	5%	1%
Partial response %	27%	29%	10%

Hazard ratio * (Pembrolizumab compared to ipilimumab) based on the stratified Cox proportional hazard model.

**Table 8:** Efficacy Results of Pembrolizumab in Ipilimumab-Refractory Melanoma.

Efficacy	Pembrolizumab 2 mg/kg Every 3 Weeks *n* = 180	Pembrolizumab 10 mg/kg Every 3 Weeks *n* = 181	Chemotherapy *n* = 179
**Progression-Free Survival**			
Number of events, *n* (%)	129 (72%)	126 (70%)	155 (87%)
Progression, *n* (%)	105 (58%)	107 (59%)	134 (75%)
Death, *n* (%)	24 (13%)	19 (10%)	21 (12%)
Median in months (95% CI)	2.9 (2.8, 3.8)	2.9 (2.8, 4.7)	2.7 (2.5, 2.8)
*p*-Value (stratified log-rank)	<0.001	<0.001	–
Hazard ratio * (95% CI)	0.57 (0.45, 0.73)	0.50 (0.39, 0.64)	–
**Objective Response Rate**			
ORR, *n*% (95% CI)	21% (15, 28)	25% (19, 32)	4% (2, 9)
Complete response %	2%	3%	0%
Partial response %	19%	23%	4%

Hazard ratio * (KEYTRUDA compared to chemotherapy) based on the stratified Cox proportional hazard model.

**Table 9:** Efficacy Results of Pembrolizumab for NSCLC.

Endpoint	*N* = 61
Overall Response Rate	
ORR %, (95% CI)	41% (29,54)
Complete response	0%
Partial response	41%

In a separate subgroup of 25 patients with limited follow-up with PD-L1 expression, TPS greater than or equal to 50% receiving pembrolizumab at a dose of 2 mg/kg every 3 weeks in Trial 1, activity was also observed. The ORR and duration of response were similar regardless of schedule (every 2 weeks or every 3 weeks) and thus the data below are pooled.

**Table 10:** Adverse Reactions of Pembrolizumab.

Immune-Mediated Adverse Reactions	Melanoma	NSCLC
Pneumonitis	2.00%	3.50%
Colitis	2.00%	0.70%
Hepatitis	1.00%	–
Endocrinopathies	0.80%	0.20%
Hyperthyroidism	3.30%	1.80%
Hypothyroidism	8.10%	6.90%
Type 1 Diabetes Mellitus	0.1%	
Nephritis	0.40%	–

**Table 11:** The New Drug Application of PD-1 Antibody in China.

Acceptance Number	Drug	Date	Company	Progress
JXSL1600007	Nivolumab	17 February 2016	Bristol-Myers Squibb	In Assessing
JXSL1500068		7 December 2015		
JXSL1300032		20 May 2013		In Clinic
JXSL1600009	Pembrolizumab	29 February 2016	Merck	In Assessing
JXSL1600005		16 February 2016		
JXSL1500074		31 December 2015		
JXSL1500058		30 September 2015		
JXSL1500040		28 July 2015		
JXSL1500020		25 May 2015		
CXSL1400138	JS001-PD-1	21 January 2015	ShangHai JunShi	In Clinic
CXSL1400153	SHR-1210	19 January 2015	ShangHai HengRui	
CXSL1500096	BGB-317	11 December 2015	BeiGene	In Assessing
CXSL1600016	Genor PD-1 Antibody	7 April 2016	Genorbio	

**Table 12:** The Clinical Phase of Avelumab.

Phase I	Phase II	Phase III
Solid Tumors	Merkel Cell Carcinoma	Non-Small Cell Lung Cancer
Renal Cancer	Non-Small Cell Lung Cancer	Renal Cell Cancer
Advanced Cancer		Gastric Cancer
Non-Small Cell Lung Cancer		Ovarian Cancer
Hodgkins Lymphoma		Urothelial Cancer
Merkel Cell Polyomavirus Infection; Stage IV Merkel Cell Carcinoma		

**Table 13:** The Clinical Phase of MEDI4736.

Phase I	Phase II	Phase III
Ovarian Cancers	Ovarian Cancers	Head and Neck Cancer
Breast	Breast	Non-Small Cell Lung Cancer
SCLC	SCLC	Breast Cancer
Gastric Cancers	Gastric Cancers	Bladder Cancer
Pancreatic Ductal Carcinoma	Pancreatic Ductal Carcinoma	Squamous Cell Lung Carcinoma
Non-Small Cell Lung Cancer	Malignant Mesothelioma	
Myelodysplastic Syndrome	Melanoma	
Advanced Solid Tumors	Hepatocellular Carcinoma	
Melanoma	Advanced Solid Tumors	
Gastric or Gastroesophageal Junction Adenocarcinoma	Glioblastoma	
Hepatocellular Carcinoma	Non-Small Cell Lung Cancer	
Head and Neck Cancer	Gastric or Gastroesophageal Junction Adenocarcinoma	
Colorectal Cancer	Colorectal Cancer	
Prostate Cancer	Esophageal Cancer	
Renal Cell Carcinoma	Sarcoma	
Malignant Mesothelioma	Mesothelioma	
Follicular Lymphoma	Lymphoma or Chronic Lymphocytic Leukemia	
Diffuse Large B-Cell Lymphoma	Myelodysplastic Syndromes	
Bladder Cancer	Oesophago-gastric Cancer	

**Table 14:** The Clinical Phase of MPDL3280A.

Phase I	Phase II	Phase III
Diffuse Large B-Cell Lymphoma, Lymphoma, Follicular		Non-Squamous Non-Small Cell Lung Cancer
Renal Cell cancer		Non-Small Cell Lung Cancer
Breast cancer	Bladder Cancer	Renal Cell Carcinoma
Bladder cancer	Advanced Non-Clear Cell Kidney Cancer	Metastatic Breast Cancer, Triple Negative Breast Cancer
Non-small cell lung cancer	Non-small cell lung cancer	Invasive Ductal Breast Carcinoma
Lymphoma	Lymphoma	Bladder Cancer
Malignant Melanoma	Colorectal Cancer	
Myelodysplastic Syndrome	Ovarian Neoplasms	
Multiple Myeloma		
Prostate Cancer		
Head and Neck Cancer		
Colorectal Cancer		

**Table 15:** The Monoclonal Antibodies of PD-1 and PD-L1.

Target	Agent	Sponsor	Class	Clinical Testing Phase
PD-1	Nivolumab	Bristol-Myers Squibb	Human IgG4	FDA-approved for treatment of refractory unresectable melanoma , for metastatic NSCLC and advanced renal cell carcinoma
	Pembrolizumab	Merck	Humanized IgG4	FDA-approved for treatment of refractory unresectable melanoma and for metastatic NSCLC that expresses PD-1
	CT-011	CureTech	Humanized IgG1k	Phase 1–2
	AMP-224	Amplimmune	PD-L2 IgG2a fusion protein	Phase 1
	MEDI0680 (AMP-514)	Amplimmune	PD-L2 fusion protein	Phase 1–2
	REGN2810	Regeneron	Human IgG4	Phase 1
	PDR001	Novartis	Information not available	Phase 1–2
	JS001-PD-1	ShangHai JunShi		
	SHR-1210	ShangHai HengRui		
	BMS-936559	Bristol-Myers Squibb	Human IgG4	Phase 1–2
PD-L1	MEDI4736	MedImmune/AstraZeneca	Humanized IgG1k	Phase 1–3
	MPDL3280A	Roche	Human IgG1k	FDA-approved for treatment of urothelial carcinoma
	MSB0010718C	Merck Serono	Human IgG1	Phase 1–3

## Abbreviations

PD-1	Programmed cell death protein 1
PD-L1	Programmed death-ligand 1
FDA	Food and Drug Administration
mAbs	Monoclonal antibodies
DCs	Dendritic cells
NK	Natural killer
NKT	Natural killer T
ITIM	Immune tyrosine-based inhibitory motif
ITSM	Inhibitory tyrosine-based switch motif
Ig	Immune globin
IFN-γ	Interferon-γ
TNF-α	Tumor necrosis factor-α
VEGF	Vascular endothelial growth factor
GM-CSF	Granulocyte-macrophage colony-stimulating factor
IL	Interleukin
PENT	Phosphatase and tensin homolog
Treg	Regulatory T cells
TIL	Tumor-infiltrating lymphocytes
ZAP	Zeta-chain-associated protein
CTLs	Cytotoxic T lymphocytes
APCs	Antigen presenting cells
MHC	Major histocompatibility complex
ADCC	Antibody-dependent cellular toxicity
NSCLC	Non-small cell lung cancer
